# Preservation of Contractile Reserve and Diastolic Function by Inhibiting the NLRP3 Inflammasome with OLT1177^®^ (Dapansutrile) in a Mouse Model of Severe Ischemic Cardiomyopathy Due to Non-Reperfused Anterior Wall Myocardial Infarction

**DOI:** 10.3390/molecules26123534

**Published:** 2021-06-09

**Authors:** Joseph Aliaga, Aldo Bonaventura, Eleonora Mezzaroma, Yogesh Dhakal, Adolfo Gabriele Mauro, Antonio Abbate, Stefano Toldo

**Affiliations:** 1 VCU Pauley Heart Center, Virginia Commonwealth University, Richmond, VA 23298, USA; aliagajs@alumni.vcu.edu (J.A.); aldo.bonaventura@vcuhealth.org (A.B.); dhakaly@vcu.edu (Y.D.); adolfo.mauro@vcuhealth.org (A.G.M.); antonio.abbate@vcuhealth.org (A.A.); 2 Department of Pharmacotherapy and Outcome Studies, Virginia Commonwealth University, Richmond, VA 23298, USA; emezzaroma@vcu.edu

**Keywords:** acute myocardial infarction, inflammation, inflammasome, NLRP3, heart failure

## Abstract

Interleukin-1β (IL-1β), a product of the NLRP3 inflammasome, modulates cardiac contractility and diastolic function. We proposed that OLT1177^®^ (dapansutrile), a novel NLRP3 inhibitor, could preserve contractile reserve and diastolic function after myocardial infarction (MI). We used an experimental murine model of severe ischemic cardiomyopathy through the ligation of the left coronary artery without reperfusion, and after 7 days randomly assigned mice showing large anterior MI (>4 akinetic segments), increased left ventricular (LV) dimensions ([LVEDD] > 4.4 mm), and reduced function (LV ejection fraction < 40%) to a diet that was enriched with OLT1177^®^ admixed with the chow in the diet at 3.75 g/kg (Group 1 [*n* = 10]) or 7.5 g/kg (Group 2 [*n* = 9]), or a standard diet as the no-treatment control group (Group 3 [*n* = 10]) for 9 weeks. We measured the cardiac function and contractile reserve with an isoproterenol challenge, and the diastolic function with cardiac catheterization at 10 weeks following the MI surgery. When compared with the control (Group 3), the mice treated with OLT1177 (Group 1 and 2) showed significantly greater preservation of their contractile reserve (the percent increase in the left ventricular ejection fraction [LVEF] after the isoproterenol challenge was +33 ± 11% and +40 ± 6% vs. +9 ± 7% in the standard diet; *p* < 0.05 and *p* < 0.005 for Group 1 and 2, respectively) and of diastolic function measured as the lower left ventricular end-diastolic pressure (3.2 ± 0.5 mmHg or 4.5 ± 0.5 mmHg vs. 10.0 ± 1.6 mmHg; *p* < 0.005 and *p* < 0.009 respectively). No differences were noted between the resting LVEF of the MI groups. These effects were independent of the effects on the ventricular remodeling after MI. NLRP3 inflammasome inhibition with OLT1177^®^ can preserve β-adrenergic responsiveness and prevent left ventricular diastolic dysfunction in a large non-reperfused anterior MI mouse model. OLT1177^®^ could therefore be used to prevent the development of heart failure in patients with ischemic cardiomyopathy.

## 1. Introduction

The NLRP3 (NACHT, leucine-rich repeat, and pyrin-domain containing protein 3) inflammasome is an intracellular macromolecular structure involved in sensing danger signals, activating caspase-1, and the pyroptotic release of pro-inflammatory cytokines that initiate the sterile inflammatory response to injury that has been identified as a central mechanism in both the response to damage and the healing process of acute myocardial infarction (MI) [1,2,3,4].

Interleukin-1β (IL-1β) is a cytokine that is produced and released by the cells due to the activation of the NLRP3 inflammasome [1,2,3,4]. As a prototypical pro-inflammatory cytokine with potent pyrogenic effects, IL-1β is responsible for the amplification of the inflammatory response as well as negatively modulating cardiac function [4,5,6,7]. Therefore, in vivo studies investigating the inhibition of IL-1β in experimental models of MI using targeted anti-inflammatory interventions have resulted in reduced adverse remodeling and progression to heart failure (HF) [8,9,10,11], which have led to human clinical trials showing promising results in patients with MI [12,13,14].

Treatment before an MI and before cardiac dysfunction ensues is typically not possible, and unfortunately, patients may already show signs of HF as they are waiting for treatment. In order to address these concerns, multiple experimental studies are investigating the efficacy of utilizing pharmacological strategies to mitigate NLRP3-modulated inflammatory signals that promote further myocardial injury in cardiovascular diseases. In a mouse model of HF due to large non-reperfused anterior MI, IL-1β blockade preserved contractile reserve and diastolic function even after the adverse remodeling of the left ventricle had ensued [8]. Consequently, the benefit of IL-1β blockade in this model paved the way to clinical trials of patients with established HF, in which the IL-1 blockers appear to improve cardiorespiratory fitness, a surrogate for contractile and diastolic reserve [15,16,17,18].

In addition to studies investigating the anti-inflammatory effects resulting from the targeting of the IL-1 receptor, IL-1β and IL-6, several NLRP3 inhibitors, which are upstream mediators of the neutralizing biologics, are under development in a variety of experimental conditions to target and inhibit the upstream NLRP3 inflammasome formation process, thereby resulting in less caspase-1 being activated, less IL-1β being produced, and an overall decrease in the sterile inflammatory response.

A recently developed small molecule NLRP3 inflammasome inhibitor, OLT1177^®^, also known as dapansutrile (3-methanesulfonyl-propionitrile), is effective in in vitro and in vivo studies, and is currently being evaluated to help treat acute gout flares, Schnitzler’s syndrome, Alzheimer’s disease, cardiovascular diseases and others [19,20,21,22]. OLT1177^®^ has been shown to be safe in humans, and it became the first NLRP3 inhibitor to complete two human proof of concept studies, one in acute gout flares (Phase 2a) and one in stable systolic heart failure (NYHA II-III) (Phase 1b) [23,24,25]. 

In the present study, we tested whether the chronic administration of OLT1177^®^ formulated in the food pellets could preserve contractile reserve and diastolic function after a myocardial infarction (MI) induced by the permanent ligation of the left main coronary artery. This experimental model was used to establish a severe ischemic cardiomyopathy that, in this mouse strain, is associated with the development of HF.

## 2. Results

### 2.1. Surgical Mortality

All of the sham-operated mice survived 10 weeks. In the MI group, the 7-day survival before treatment allocation was 65% (35 on a total of 54 undergoing surgery). Six mice were classified as screen failures following the 3-day screening echocardiography, and thus, excluded due to presenting either small infarctions (less than four affected LV segments) or LVEDD < 4.4 mm. After the assessment of the screening echocardiogram, 29 mice meeting the criteria for severe adverse left ventricular remodeling were assigned to the four different groups, each with different diets, by an investigator not involved in the assessment of the endpoints: 10 mice were assigned to Group 1—OLT1177^®^, 3.75 g/kg diet; 9 mice were assigned to Group 2 OLT1177^®^, 7.50 g/kg diet; 10 mice were assigned to Group 3, standard chow diet. The six shams were assigned to Group 4, i.e., standard chow diet. At the end of 10 weeks, 7 of the 29 (24%) mice with AMI had died: two mice from Group 1, two mice from Group 2, and three mice from Group 3. None of the mice with the sham operation died within 10 weeks. 

### 2.2. Diet and Weight

**Figure 1:**
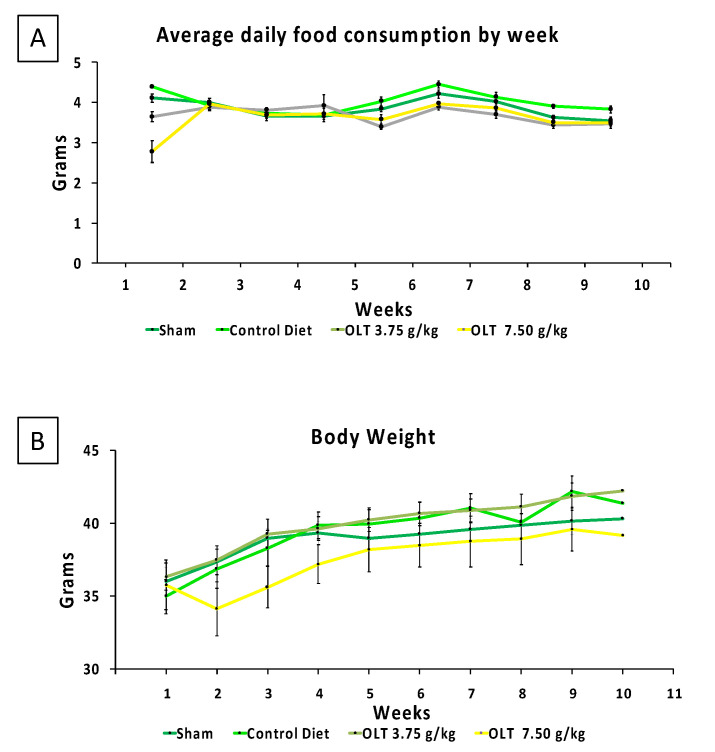
(**A**,**B**) The diets were administered seven days post-AMI surgery, and the average daily food consumption (**A**) and corresponding body weight (**B**) of the mice groups were measured and recorded during a 9-week experimental period. The values are expressed as the mean ± SEM.

Figure 1 shows a weekly measurement of diet consumption and weight gain. Of the four groups, Group 2 (7.50 g/kg OLT1177^®^ diet) consumed considerably lower amounts of their diet with the corresponding body weight during the first week, as compared with the other diets. By week 2, the diet consumption was not different when comparing the different groups, and it remained not different until the end of the follow up. 

### 2.3. Cardiac Remodeling

**Figure 2:**
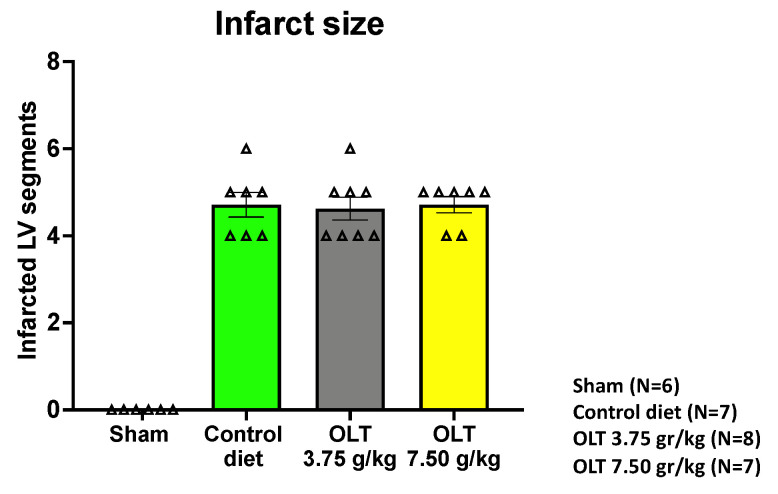
Assessment of the size of the infarct using transthoracic echocardiography 3 days after MI. Only the mice with four or more infarcted left ventricular segments out of a total of 16 (six segments at the base, six mid-ventricular segments, and four apical segments) were included in the study. Each triangle corresponds to the value measured in each mouse. The values are expressed as the mean ± SEM.

The data analysis included all of the mice that survived from the moment of the diet randomization to the end of the study. The number of infarcted segments in each group, as assessed by echocardiography, is reported in Figure 2. 

**Figure 3:**
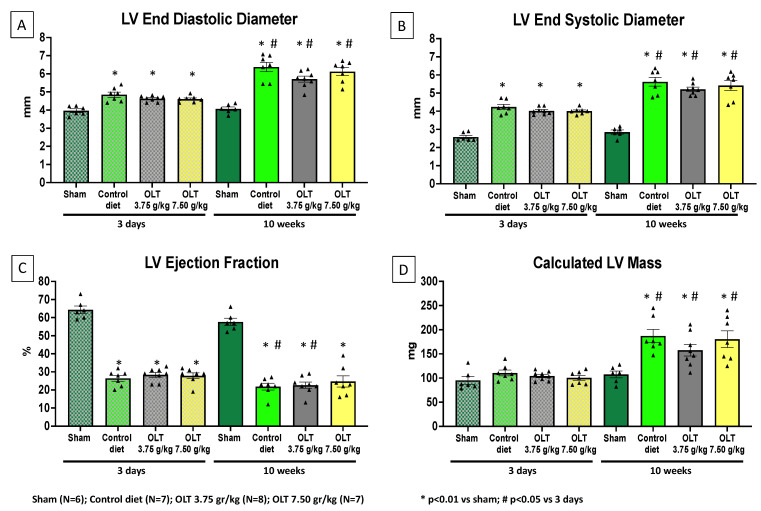
Effects of OLT1177 on left ventricular function and remodeling. The echocardiographic results at 3 days and 10 weeks post-MI surgery indicate that no significant differences were reported between the control group and both OLT-treated groups. (**A**,**B**) LVEDD and LVESD significantly increased in the control group and the OLT-treated mice. (**C**) The LVEF values recorded show a significant reduction in the control group and the OLT-treated mice. (**D**) The LV mass of the control group and of both OLT-treated groups also show a significant increase throughout the study. Each triangle corresponds to the value measured in each mouse. The values are expressed as the mean ± SEM.

As expected, at the baseline, when compared to the sham-operated mice (*n* = 6), the mice that underwent AMI surgery had an 18% greater LVEDD (*p* < 0.001) and a 57% lower LVEF (*p* < 0.001). After 10 weeks, a further increase in LVEDD and decrease in LVEF was noted in the mice fed with the standard diet (Group 3), as well as in those fed with low- and high-dose OLT1177^®^ in the diet (Groups 1 and 2, respectively), without significant differences between the groups (Figure 3).

### 2.4. Contractile Reserve

**Figure 4:**
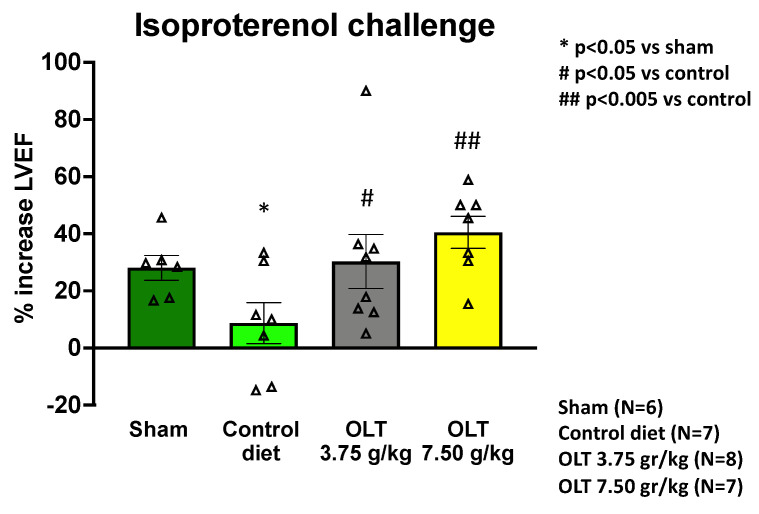
LVEF contractile reserve assessment at 10 weeks after the non-reperfused MI surgery shows that the mice treated with OLT1177 (3.75 g/kg and 7.50 g/kg) had a significant % increase in LVEF following the isoproterenol challenge, reflecting an increase in the β-adrenergic response/contractile reserve. Each triangle corresponds to the value measured in each mouse. The values are expressed as the mean ± SEM.

The left ventricular contractile reserve is the difference between the contractility of the LV at rest compared to stress. In our study, it was measured as the percent change in LVEF after the isoproterenol challenge. Ten weeks after MI, the mice fed with low- or high-dose OLT1177^®^ diet (Groups 1 or 2) displayed significantly greater preservation of their contractile reserve than the mice fed with the control diet (Group 3) (+33 ± 11% or +40 ± 6%, respectively, versus +9 ± 7%, *p* = 0.034, and *p* = 0.002), while also displaying no significant difference from the sham-operated mice (Group 4) (+28 ± 4%, *p* = 0.750, and *p* = 0.117) (Figure 4). No difference was noted between the groups fed with the two active diets.

### 2.5. Diastolic Function

**Figure 5:**
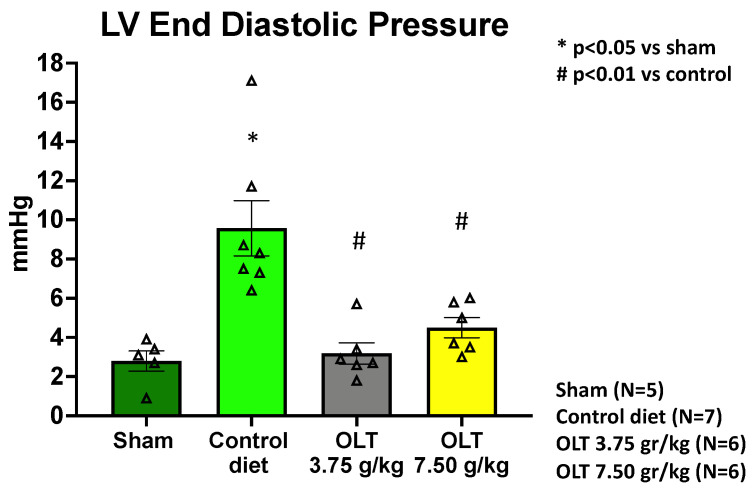
LV catheterization and assessment of LVEDP at 10.5 weeks after the coronary artery ligation surgery show that both of the OLT-treated mouse groups (3.75 g/kg and 7.50 g/kg) had a significant decrease in LVEDP, indicating that the treatment with OLT1177 led to a preserved diastolic function. Each triangle corresponds to the value measured in each mouse. The values are expressed as the mean ± SEM.

LVEDP was measured between 48 and 72 h after the isoproterenol challenge, a time that ensures the clearance of the short-acting β-adrenergic agonist. The results from the LV catheterization show a significantly lower LVEDP in the mice fed with low- and high-dose OLT1177 diets (Groups 1 or 2) when compared to the mice on the control diet (Group 3) (3.2 ± 0.5 mmHg or 4.5 ± 0.5 mmHg, respectively, versus 9.6 ± 1.4 mmHg in; *p* = 0.004 and *p* = 0.009), while also displaying a small but significant difference from the sham-operated mice (Group 4) at the higher dose (2.8 ± 0.5 mmHg, *p* = 0.627 vs. Group 1 and *p* = 0.047 vs. Group 2) (Figure 5).

## 3. Discussion

We studied the effects of the selective NLRP3 inhibitor, dapansutrile (OLT1177^®^), on a mouse model of severe systolic heart failure in which a 9-week oral treatment period began 7 days after MI, and herein show for the first time that the chronic inhibition of NLRP3 improves cardiac contractile reserve and preserves diastolic function after the acute healing process has occurred.

In the presence of ischemia associated with a myocardial injury, cell death and cell debris act as damage associated molecular patterns (DAMPs) that are sensed by NLRP3 and induce its activation. Following the oligomerization and assembly of the active NLRP3 inflammasome, pro-caspase-1 is recruited and activated to process and release the pro-inflammatory mediators IL-1β and IL-18. Several reports have demonstrated that the extracellular release of these proinflammatory cytokines leads to an excessive or dysregulated form of inflammation characterized by increased inflammatory cell death (pyroptosis), the additional loss of functional myocardium, and further dysregulation in the recovery processes (fibrotic non-functional scar formation) that can promote more advanced systolic and diastolic dysfunction and contribute to the development of HF [1,5,6,7,26]. The potential of attenuating the inflammatory response in cardiovascular diseases has led to studies investigating anti-inflammatory approved anti-IL-1β interventions [2,7,8,12]. 

These previous studies investigating an IL-1 receptor antagonist or an IL-1β monoclonal antibody, such as anakinra and canakinumab, respectively, have demonstrated that IL-1β blockade prevents and partially reverses cardiac diastolic and systolic dysfunction in a variety of animal models, as well as being associated with a reduced incidence of HF and HF hospitalizations in patients with AMI [8,14,27].

With the validation by these biologics, NLRP3 inhibitors under development have been shown to preserve cardiac function in ischemic injury animal models [28]. Furthermore, a recent study investigating the effects of an NLRP3 inhibitor, OLT1177^®^ (dapansutrile), demonstrated that—in an experimental mouse model of AMI due to myocardial ischemia-reperfusion injury—OLT1177^®^ (6, 60, or 600 mg/kg i.p.), when administered within 60 min following ischemia-reperfusion injury, significantly inhibited the caspase-1 activity in the heart, preserved the cardiac contractile function, and reduced the size of the infarct. These results support the potential clinical translational value of utilizing OLT1177^®^ as a cardioprotective strategy after myocardial injury [29].

The present study builds on our prior study by investigating the effects that NLRP3 inhibition could have on mitigating aberrant NLRP3 pro-inflammatory signaling in a mouse model of ischemic heart failure induced via permanent coronary artery occlusion. This mouse model has been extensively used and shows signs of HF in mice, including a severe reduction of ejection fraction, impaired contractile reserve, lung congestion, and increased filling pressure at rest [30,31]. In our study, one week following the non-reperfused MI and after the healing process began, OLT1177^®^ was administered to the mice with severe injuries (at least 4/16 akinetic LV segments and LVEDD > 4.4 mm) and was continued for 9 weeks. In contrast to the prior studies investigating anti-inflammatory interventions targeting downstream NLRP3 mediators of inflammation (IL-1 receptors, IL-1β, IL-6), this present study was designed to utilize OLT1177^®^ as a selective NLRP3 inhibitor of which the mechanism of action targets the NLRP3 ATPase activity that contributes to the NLRP3 oligomerization of the NLRP3 inflammasome [32], thereby resulting in less caspase-1 being activated and less IL-1β being produced, thus preserving the contractile reserve and diastolic function following a non-reperfused myocardial infarction [20,28]. 

The contractile reserve depends on the integrity of the cardiomyocytes as well as the adrenergic receptor density on the cardiomyocyte surface [33]. In HF, the myocardial sensitivity to catecholamines is reduced, leading to reduced contractility and the reduced adaptation to increased myocardial workload, e.g., during exercise. Therefore, a good contractile reserve is quite an important phenotype in HF. The data collected in this current study have shown that NLRP3 inhibition with OLT1177^®^ has a potential therapeutic value in preserving contractile reserve/β-adrenergic responsiveness, by way of preventing the significant desensitization and/or downregulation of the β-adrenergic receptors responsible for impairing the contractile reserve, a distinctive feature of cardiomyopathy and HF [34]. We believe that the mechanism by which OLT1177^®^ improved the β-adrenergic responsiveness to isoproterenol is through the reduction of IL-1β production. Prior studies have shown that recombinant IL-1β reduces the response to isoproterenol in mice [6,35]. In addition, IL-1 blockade improved the isoproterenol response in mice treated with human plasma from patients with acute decompensated HF and high CRP, suggesting that high IL-1 activity impairs the contractile reserve in these mice [35]. In the mouse model of permanent coronary artery occlusion, IL-1β inhibition for 3 days, initiated 9.5 weeks after MI, is sufficient to increase the contractile reserve, confirming again an important role of IL-1 in affecting β-adrenergic responsiveness [8]. OLT1177^®^ for 9 weeks also showed a potential therapeutic value in preserving the left ventricular diastolic function following MI. The effects of OLT1177^®^ on contractile reserve and LVEDP were independent of any effect on cardiac remodeling after MI. Our results suggest that the effects of NLRP3 inflammasome activity affect cardiomyocyte function, and that the outcomes resulting from chronic OLT1177^®^ may be applicable to the most severe forms of HF. Recently, a phase IB safety study in patients with chronic systolic HF showed that OLT1177^®^, dapansutrile— given for 14 days—was well-tolerated and safe, and, in the cohort receiving the highest dose (2000 mg daily), it was associated with an improvement in exercise time on the treadmill and LVEF [25]. The findings with OLT1177^®^ are also consistent with previous studies that have investigated the inhibition of IL-1β signaling and shown it to preserve contractile reserve [6,8]. In a similar model of non-reperfused MI, IL-1β blockade showed analogous results regarding the significant preservation of diastolic function and the restoration of the contractile reserve, while having no significant effect on cardiac remodeling [8]. 

This study has several limitations. First, is a limitation often seen in preclinical studies involving the use of mice, such as using a single animal breed, using only relatively young healthy male mice (i.e., free of cardiovascular risk factors and comorbidities), and the obvious differences with humans. A second limitation pertains to the conditions seen in patients with a non-reperfused MI that cannot be thoroughly reproduced in an experimental animal model. Third, is the simulation of a consistent method of administering OLT1177^®^ through the diet, wherein the study subjects’ differences in acclimatizing to their new diet and food preferences can make it difficult to ensure that the appropriate therapeutic range of the experimental drug is being met on a consistent basis without measuring the test subject’s blood/plasma regularly. We explored two oral doses that have been tested and were proven to be effective in different mouse models of disease (Alzheimer’s and cancer) [22,36]. It is therefore possible that a higher dose is necessary to block the residual remodeling happening between week 2 and week 10, and to improve the resting LVEF. Fourth, we did not assess the chronic signs of HF in our mice (e.g., lung congestion). However, prior reports have shown that the MI model we used induces HF in mice [30,31]. Fifth, we did not use histological techniques to measure the scar size. We used ultrasounds as a method to quantify the infarcts at 3 days after MI. We have previously shown that this method closely correlates with the histological analysis [37]. In addition, we do not expect OLT1177^®^ to reduce the scar size in this model of MI. In fact, NLRP3 inhibition or gene deletion failed to reduce the scar size in this model of non-reperfused MI [26,28,38]. 

## 4. Materials and Methods 

### 4.1. Ethical Approval

All of the experimental procedures were performed in accordance with the “Guide for the Care and Use of Laboratory Animals” published by the National Institutes of Health (8th ed. revised 2011). The study protocol was approved by the Virginia Commonwealth University Institutional Animal Care and Use Committee.

### 4.2. Study Design

**Figure 6:**
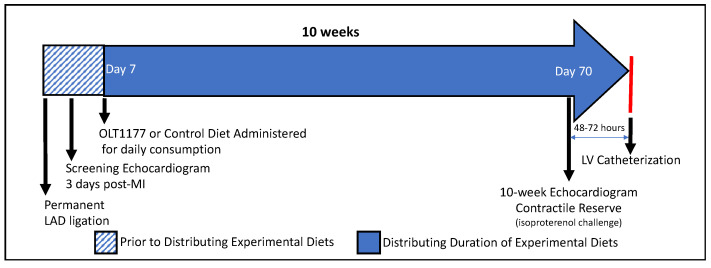
The study was designed to assess cardiac function and contractile reserve following an experimental murine model of the ligation of the left coronary artery without reperfusion in mice consuming an OLT1177-enriched diet. (1) Three days after MI surgery, all of the mice underwent transthoracic echocardiography and were randomly assigned into four groups. (2) After their recovery from the surgery, the four groups of mice were administered either a standard diet or an OLT1177-enriched diet (3.75 g/kg or 7.50 g/kg). (3) Seventy days post-MI, the effects of OLT1177 on cardiac function and contractile reserve were assessed via echocardiography and isoproterenol challenge. (4) About 2–3 days after the 10-week echocardiography and contractile reserve assessments, the diastolic function of all of the surviving mice was assessed via LV catheterization. Abbreviations: LAD = left anterior descending coronary artery; AMI = acute myocardial infarction; SD = standard diet; LV = left ventricular.

The objective of this study was to examine whether OLT1177^®^ preserved or restored the contractile reserve and diastolic function in mice with severe adverse left ventricular remodeling and ischemic heart failure secondary to a large non-reperfused anterior MI. In brief, all of the mice were subjected to either an experimental MI via the permanent ligation of the left main coronary artery or a sham operation, as previously described [4,28]. Three days after the surgery, the surviving mice underwent transthoracic echocardiography (TTE) assessment. The mice with a large non-reperfused anterior infarct [>4 akinetic segments], dilated left ventricle [LV] (LV end-diastolic diameter [LVEDD] > 4.4 mm), and systolic dysfunction (LV ejection fraction [LVEF] < 40%) were randomly assigned to one of the following groups: chow enriched with OLT1177^®^ 3.75 g/kg after MI (Group 1), chow enriched with OLT1177^®^ 7.50 g/kg after MI (Group 2), a standard chow diet after MI (Group 3), and a standard chow diet after sham surgery (Group 4). The mice then underwent the evaluation of their cardiac function and contractile reserve 10-weeks post-AMI surgery, as well as the assessment of their diastolic function by cardiac catheterization prior to their sacrifice. A timeline of the study is shown in Figure 6.

### 4.3. Experimental AMI Model 

We used 60 adult male ICR mice (8–10 weeks old, 35–45 g body weight) supplied by Envigo (Indianapolis, IN) and subjected them to an open-heart surgical procedure. Of these, 54 mice underwent an experimental AMI procedure, while the remaining 6 mice underwent a sham operation procedure, as previously described [4,8,11,28]. Briefly, a single operator performed all of the procedures with the aid of an operating microscope (Leica). The left anterior descending coronary artery was permanently ligated near its origin with a 7.0 silk suture. The same procedure without the ligation was performed in the sham group. This non-reperfused AMI model was utilized to simulate the severe changes that occur following a myocardial injury, such as adverse cardiac remodeling in the form of ischemic dilated cardiomyopathy and LV hypertrophy [26]. The mice were anesthetized with a peritoneal injection of sodium pentobarbital (50–70 mg/kg), shaved in the left chest area, secured in a supine position, and orotracheally intubated by being connected to a positive-pressure ventilator with a tidal volume set at 0.25 mL, and with the respiratory rate adjusted to 133 cycles/min. After each coronary artery occlusion or sham operation, the thoracic cavity was closed with sutures. Once spontaneous respiration resumed, the orotracheal tube was removed and the mice were left to recover from the anesthesia on heating pads, and slow-release buprenorphine (1 mg/kg) was administered. The supervision of the animals continued after surgery, and once fully awake, the mice were housed under climate-controlled conditions in a 12-hour light/dark cycle and provided with standard mouse chow and water ad libitum for the next 7 days, wherein afterward their diet was changed to either the standard diet or the diet containing low- or high-dose OLT1177^®^.

### 4.4. Treatment 

OLT1177^®^, a pharmacological inhibitor of NLRP3, was formulated into mice chow (0.5″ pellets) and color-coded by Research Diets, Inc. (New Brunswick, NJ, USA). Two OLT1177^®^ diets were prepared with either low- or high-dose OLT1177^®^ (3.75 g or 7.50 g of OLT1177^®^ per kg of food) and administered starting 1 week after AMI surgery in Groups 1 and 2 for the entire duration of the study. Research Diets, Inc. (New Brunswick, NJ, USA) also provided the control chow, which was labeled as the standard/control diet. This control diet did not contain OLT1177^®^, and was administered to Group 3 (the control group mice) and Group 4 (the sham-operated mice). The mice were randomly assigned to different treatments by an investigator not involved in the assessment of the endpoints following TTE assessment 3-days post-AMI. Throughout the rest of this 10-week study, the survival of the animals and the average food consumption of every cage were assessed and recorded every 1–3 days. In order to ensure that the mice were eating sufficiently, food was added regularly, and further monitoring was carried out by weighing and recording the weight of each mouse once a week for the remaining 9 weeks. In addition, all of the cages were examined daily to ensure the well-being of the mice. 

### 4.5. Transthoracic Echocardiography

All of the mice underwent transthoracic echocardiography at 3 and 70 days after surgery. The echocardiograms were obtained in order to assess and monitor the change in LV systolic function, infarct size, cardiac remodeling, and wall motion abnormalities between the groups of mice. The echocardiography was performed using a Vevo770 High Resolution (VisualSonics Inc., Toronto, ON, Canada) along with a transducer probe with a high transmission frequency of 30 MHz [39]. Briefly, each mouse was sedated with sodium pentobarbital (30–50 mg/kg) (Diamondback Drugs, Scottsdale, AZ, USA), shaved at the upper and lower chest area, placed in a supine position on a warmed Vevo770 platform, and ultrasound gel was applied. Each heart was then visualized in B-mode (2-D imaging) from the parasternal short-axis and apical views, which allowed us to measure the LV end-diastolic diameter (LVEDD) and LV end-systolic diameter (LVESD) at M-mode, according to the American Society of Echocardiography recommendations [40], as previously described [9]. The left ventricular fractional shortening (LVFS) and ejection fraction (LVEF) were calculated according to the following formulas: LVFS = (LVEDD − LVESD)/LVESD × 100) averaged over several cycles, whereas the LVEF was derived using the Teicholz formula, a formula dependent on estimating LV end-systolic and end-diastolic volumes to the third power of the diameter multiplied by a factor of 1.047 according to the ellipsoid shape in which the length is twice the diameter (LVEF = [LVEDD^3^ − LVESD^3^]/LVEDD^3^), as previously described [5,6,9]. The assessment of LVFS and LVEF was performed off-line by an investigator blinded to the treatment allocation.

### 4.6. Assessment of the Contractile Reserve

We assessed the contractile reserve 70 days after the MI surgery through an echocardiogram assessment of LVEF and isoproterenol challenge. Isoproterenol (20 ng/mouse) is a synthetic short-acting β-adrenergic receptor agonist (2–5 min half-life); it was administered intraperitoneally to each anesthetized mouse in order to assess the contractile reserve/β-adrenergic sympathetic response [8,41]. LVEF was monitored continuously after isoproterenol injection and the contractile reserve was expressed as the percentage of the LVEF interval’s change from the baseline. The dose used did not alter LVEDP even when administered during the hemodynamic assessments [42]. The measurement of LVEF was performed off-line by an investigator blinded to the treatment allocation.

### 4.7. Assessment of the Left Ventricular Diastolic Pressure

Between 48 and 72 h after the isoproterenol challenge, all of the mice underwent anesthesia using pentobarbital (70 mg/kg) before LV catheterization, a terminal procedure utilized to determine the effect of the OLT1177^®^ on the left ventricular diastolic pressure, as previously described [8]. Directing a Millar Mikro-Tip^®^ Pressure Transducer Catheter (AD Instrument, Houston, TX, USA) into the right carotid artery, through the aorta, and into the left ventricle allowed the measurement and recording of the intracardiac LV pressure-volume tracings. The effect of the treatment on the left ventricular diastolic pressure was characterized by comparing the left ventricular end-diastolic pressures (LVEDP) of the experimental groups to the control group. The values (mmHg) from this pressure probe catheter were recorded in real-time and measured using Labchart Pro 5 (AD Instruments Houston, TX, USA). The LV catheterization, as well as the assessment of LVEDP, were performed by an investigator blinded to treatment allocation.

### 4.8. Statistical Analysis 

The data and values derived from these procedures and techniques are presented as the mean and the standard error of the mean. The normality of the data distribution was determined using the Kolmogorov–Smirnov test. For the differences between the mouse treatment groups, comparisons were made utilizing a two-tailed Student’s T-test, whereas comparisons of the two different doses and the control condition were assessed using one-way analysis of variance (ANOVA) followed by Bonferroni’s post-hoc test for multiple comparisons. The missing data (i.e., mortality related to procedural mortality) was excluded from our experimental results and analyses. The unadjusted *p*-values are reported throughout, with statistical significance set at the two-tailed 0.05 level (*p* < 0.05). Prism 9.1.0 (GraphPad Software LLC, San Diego, CA, USA) for Mac was used.

## 5. Conclusions

The inhibition of NLRP3-dependent IL-1β with OLT1177^®^ (dapansutrile), administered orally, preserved the contractile reserve and diastolic function in a preclinical model of severe ischemic heart failure in mice after 10 weeks of treatment. The data presented support the notion that NLRP3 inhibition with OLT1177 may improve the cardiac function and reserve of patients with HF.

## Data Availability

Single data point are presented in each figure of the study. The database will be made available upon request to the corresponding author.

## Sample Availability

Not available.
